# Supplementary figures and images for: Genome-wide comparison of four MRSA clinical isolates from Germany and Hungary
Source: PeerJ. 2021 Jan 13;9:e10185. doi: 10.7717/peerj.10185 (PMC7811285; doi:10.7717/peerj.10185)

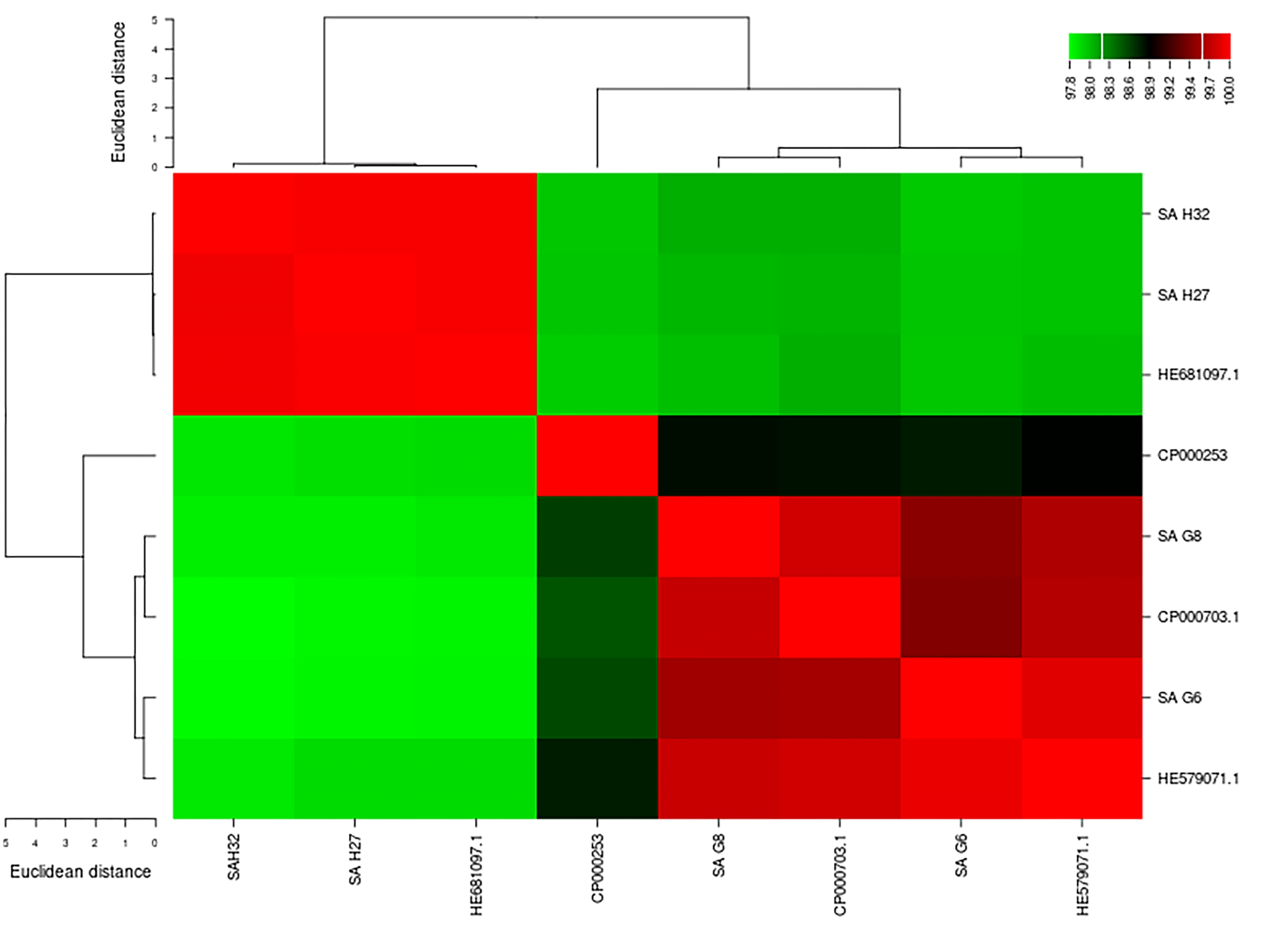

Supplement: Supplemental Information 1 [file peerj-09-10185-s001.jpg]

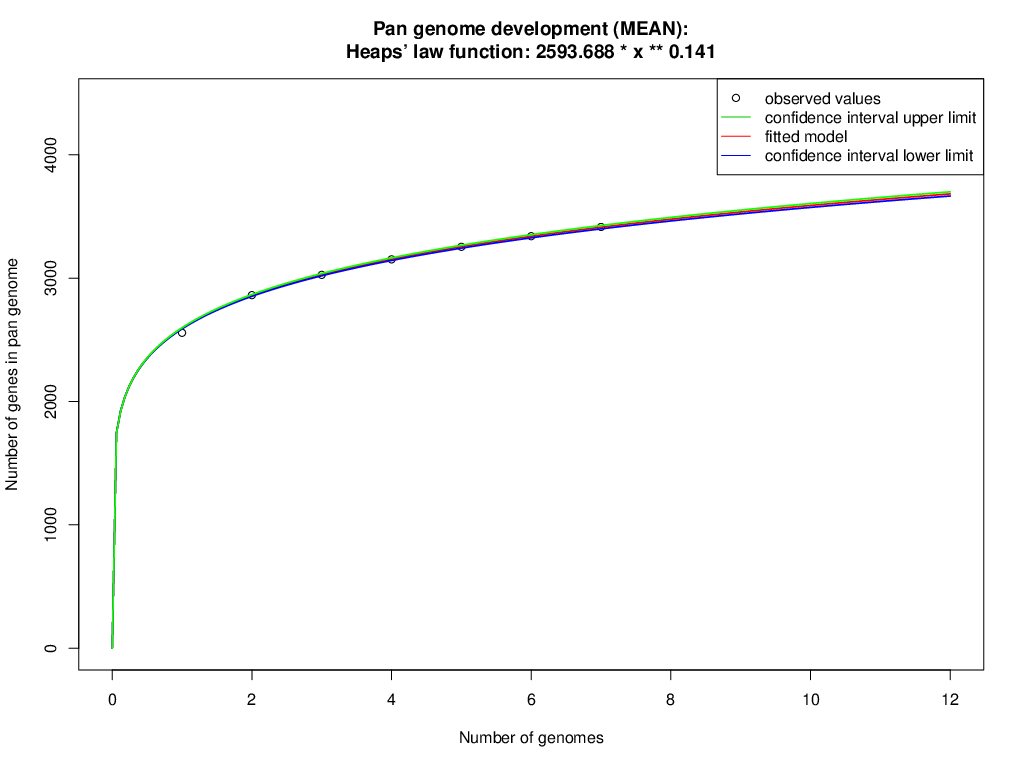

Supplement: Supplemental Information 2 — The pan-genome development analysis suggested that increases with every additional S. aureus strain, indicating an open pan-genome (Heaps’ law function: 2593.688* x **0.141). [file peerj-09-10185-s002.png]

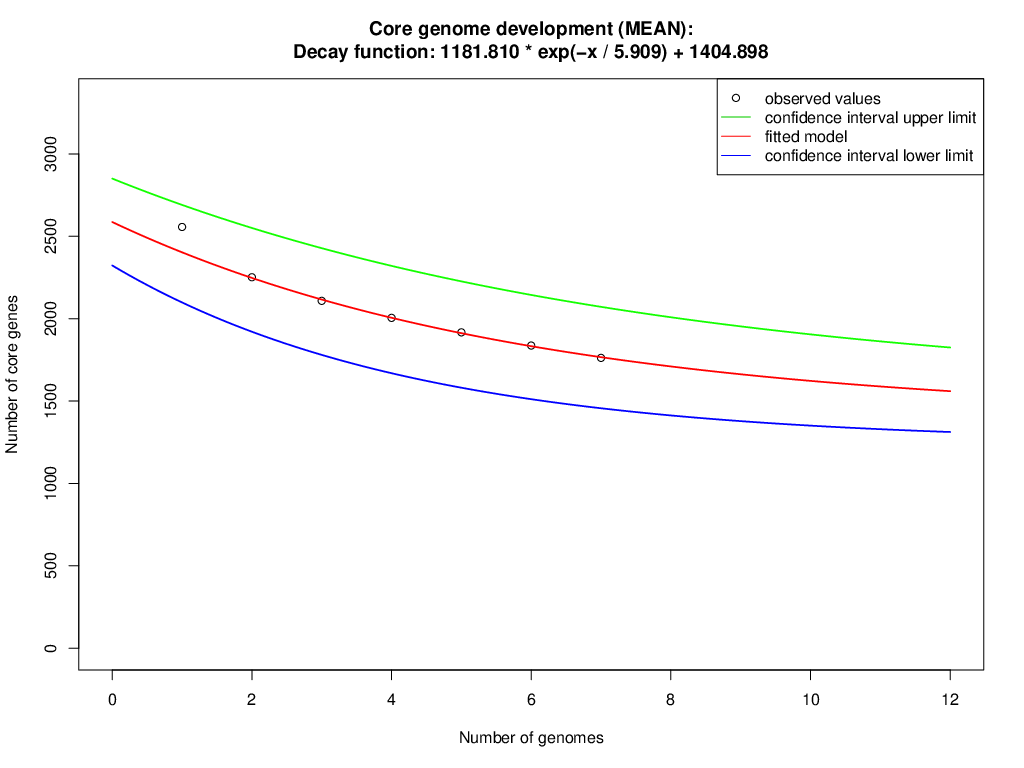

Supplement: Supplemental Information 3 — The calculated core genome will be around 1404.8 CDS, based on a decay function (1181.810* exp( −x/5.909) +1404.898). [file peerj-09-10185-s003.png]

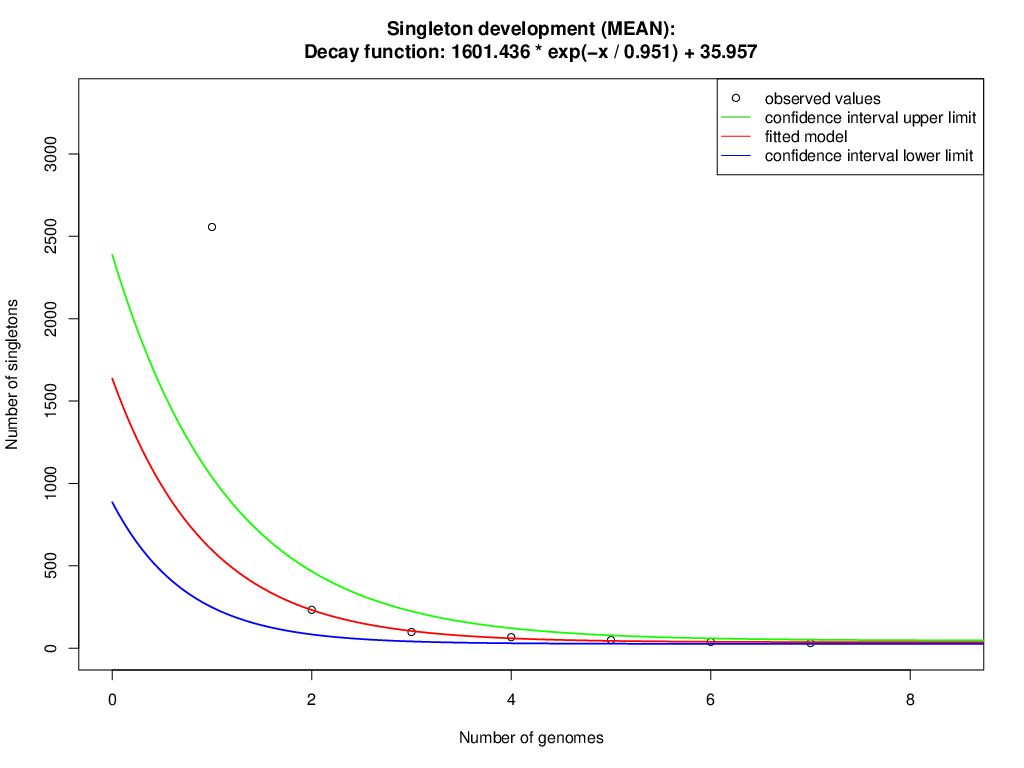

Supplement: Supplemental Information 4 — The plot suggested that the pan-genome size will continue to expand at the rate of 35.9 genes per novel, representative genome based on a decay function (1601.436* exp(-x/0.951+35.957). [file peerj-09-10185-s004.png]
