# Supplementary material for: Genome-wide comparison of four MRSA clinical isolates from Germany and Hungary
Source: PeerJ. 2021 Jan 13;9:e10185. doi: 10.7717/peerj.10185 (PMC7811285; doi:10.7717/peerj.10185)
Supplement: Supplemental Information 5 [file peerj-09-10185-s005.docx]

**Table S1:**

**The acidic and alkaline pH survival rate in percentage of *S. aureus* isolates**

| **Strains**  **pH condition** | **SA G6** | **SA G8** | **SA H27** | **SA H32** |
| --- | --- | --- | --- | --- |
| pH 4.5 | 84.20±0.02 | 84.40±0.03 | 45.19±0.02 | 73.01±0.02 |
| pH 9.5 | 78.84±0.02 | 77.35±0.04 | 79.37±0.03 | 63.43±0.05 |

Values are given as % mean ± SEM (n = 3)
