# Supplementary material for: Genome-wide comparison of four MRSA clinical isolates from Germany and Hungary
Source: PeerJ. 2021 Jan 13;9:e10185. doi: 10.7717/peerj.10185 (PMC7811285; doi:10.7717/peerj.10185)
Supplement: Supplemental Information 6 [file peerj-09-10185-s006.docx]

**Supplementary Table S1:**

**Prophage features of *S. aureus* isolates**

| **Prophage** | **Host** | **Size**  **(kb)** | **ORF** | **Gene** | **G+C%** | **Completeness** | **Lifestyle** | **Region Position** | **Virulence & resistance genes** |
| --- | --- | --- | --- | --- | --- | --- | --- | --- | --- |
| phiG6.1 | SA G6 | 16.4 | 72.8 | 26 | 33.4 | Incomplete | Temperate | 1046367-1062805 | *isdD* |
| phiG6.2 | SA G6 | 72.8 | 74.5 | 99 | 34.6 | Intact | Temperate | 1930919-2003736 | *sea, sep, sak, scn, atl* |
| phiG6.3 | SA G6 | 74.5 | 8.9 | 92 | 33.3 | Questionable | Temperate | 2753517-2828049 | *lukF-PV, lukM, b-lactamase, plc, aadA, aphA1, sta* |
| phiG8.1 | SA G8 | 8.9 | 51.4 | 17 | 35.1 | Incomplete | Temperate | 250716-259621 | *-* |
| phiG8.2 | SA G8 | 51.4 | 77.1 | 87 | 34.1 | Intact | Temperate | 659680-711162 | *ear* |
| phiG8.3 | SA G8 | 77.1 | 54.4 | 95 | 32.6 | Intact | Temperate | 1480513-1557685 | *virE, ebp* |
| phiG8.4 | SA G8 | 54.4 | 32.7 | 71 | 32.3 | Intact | Temperate | 2002216-2056682 | *lukF-PV, lukM, plc, sep, sak, chp, scn* |
| phiG8.5 | SA G8 | 32.7 | 14.6 | 39 | 36.7 | Questionable | Temperate | 2827472-2860207 | *β-lactamase* |
| phiH27.1 | SA H27 | 71.7 | 320 | 88 | 32.7 | Intact | Temperate | 1496964-1568760 | *virE* |
| phiH27.2 | SA H27 | 45.4 | 235 | 64 | 33.0 | Intact | Temperate | 2005223-2050666 | *plc, hlgB, lukF-PV, lukM, scn, chp, sak, hlb* |
| phiH27.3 | SA H27 | 16.5 | 91 | 19 | 35.0 | Intact | Temperate | 2768835-2785368 | *cna, atl* |
| phiH32.1 | SA H32 | 104.6 | 364 | 131 | 32.7 | Intact | Temperate | 1956029-2060686 | *lukF-PV, lukM, hlgB, plc* |
